# Concepts in local treatment of extensive paediatric burns

**Published:** 2014-06-25

**Authors:** M Ungureanu

**Affiliations:** "Grigore Alexandrescu" Hospital, Plastic and Reconstructive Surgery and Burns Department

**Keywords:** extensive burns, local topics, autograft, allograft, synthetic and semi-synthetic skin substitutes

## Abstract

Abstract

There is a wide variety of local therapeutical methods for extensive burns. This article aims to be a general overview of the most common methods used in the local treatment for extensive burns, both in our clinic and globally. Clinical examples are shown from our clinic; cases of the last 8 years. None of the less there is no such thing as the "perfect method of treatment" but a thin balance between the clinical experience of plastic surgeons, every case particularities and specified characteristics, meaning advantages, disadvantages and limited indications of local topics or methods of skin covering.

## Introduction

Burns are a very serious public health problem because of their large number, the gravity of lesions, their prognosis, the large number and the gravity of complications, the treatment during a long period of time, the functional, esthetic, psychological and social effects. 

 This article is referring only to extensive burns meaning burn lesion covering more than 30% of BSA. 

 The etiological agents’ incidence has a particular pattern. Scalds are more frequent meaning 62%, followed by flame 20%, electrical injury 8%, contact burns 5%, collective accidents (usually explosions) 5%. 

 In extensive burns, after hemodynamic and electrolytic resuscitation has been initiated, the main factor in ulterior evolution of the patient is represented by the local treatment of burns lesions, which has as main goals the stopping of the loss of fluids from the burn lesion itself, the prevention or better said the control of the microbial colonization, the fastening of the spontaneous re-epithelialization in cases of partial burns and the performance of escharotomy and immediate coverage for deep burns[**1,2,11**]. 

 The treatment principles are the following: determination of dimension and depth of the burn, decision upon conservative or surgical treatment viable tissues preservation (first aid: tissue cooling, dry, clean adequate wound dressings), preventing microvascular deterioration and edema reducing: positioning, early escharotomies, infections preventions (debridement, necrectomies, local and systemic antibiotic therapy), fast skin covering (necrosectomy, skin grafts, skin substitutes, skin flaps), sequelae prevention by positioning, splinting, pressure therapy, functional rehabilitation by early active and passive motion due to physiotherapy, secondary and tertiary corrections, if indicated [**1,11**].

## Material and methods

For the period of 8 years, meaning 2006-2013, 3128 burned patients were admitted in our clinic, of which 41,5% were extensive burns. 

 Local methods of burns’ treatment are synthesized below: 

 Local topics: antibacterial topics agents, topics that fasten re-epithelization, dressings with low delivery of active component, enzymatic debridement [**6,7**]. 

 Methods of skin covering: 

 -definitive skin covering: autograft, alternative materials (dermal matrix, composite materials, autologous epidermal culture) 

 -temporary skin covering: natural skin substitutes (allografts, xenografts, amniotic membranes), synthetic and semisynthetic skin substitutes (bilayered synthetic structures), composite materials based on collagen - Biobrane) [**11,21,22**]. 

 The eschar forms on both partial- and full-thickness burns. With increasingly thick eschar over deeper burns, the administration of systemically administered antibiotics to eschar is not reliable. 

 Post-burn. The eschar is virtually sterile, but in the absence of topical antimicrobials, it will be colonized in the first 24 h by Gram-positive organisms that are superseded in 3 to 7 days by Gram-negative species [**4**]. 

 Specific agents 

 None of the topical antimicrobials available today, whether alone or in combination, has the characteristics of an ideal prophylactic agent (easy to store, easy to apply, painless, good eschar penetration, no systemic absorption, not toxic, no wound healing retardation, long-lasting, inexpensive, broad spectrum of activity), but they will eliminate colonization of burn wounds, and invasive infections are infrequent [**5**]. 

 Below is a presentation of a summary of topical agents in current use. 

 Silver sulfadiazine: With an excellent spectrum of activity, low toxicity, and ease of application with minimal pain, silver sulfadiazine is still the most frequently used topical agent. 

 Mechanism of action. Silver sulfadiazine is thought to act via inhibition of DNA replication and modifications of the cell membrane and cell wall. Spectrum of antimicrobial activity. The drug is bactericidal against species of both Gram-positive and GramneLyative organisms, but resistance has occasionally been reported. Clinical use. Silver sulfadiazine is used as an adjunct in the prevention and treatment of infection in second- and third-degree burns. However, treatment fails with continued use in large burns (> 50qc TBSA). Concomitant administration of appropriated systemic anti-infective agents may be necessary if the infection is present or suspected. The use of silver sulfadiazine is frequently associated with the development of a "pseudo-eschar" within 2 to 4 days, owing to the interaction of the drug with proteinaceous exudate in the wound, which can lead to error in the evaluation of burn depth by the inexperienced observer. Adverse effects. Local skin reactions, such as pain, burning, or itching and hypersensitivity, are occasionally reported. Transient leukopenia occurs in 5 to 15% of patients, but there is no increased incidence of infectious complications. This may be an intrinsic response to burn injury unrelated to the use of silver sulfadiazine. Systemic absorption may produce reactions characteristic of sulfonamides, including crystalluria or methemoglobinemia [**1,3,11**]. 

 Cerium nitrate-silver sulfadiazine: the incorporation of cerium nitrate to sulfadiazine results in enhanced clinical efficacy in patients with large burns. Mechanism of action. Cerium nitrate has antimicrobial activity in vitro and reverses post-burn cell-mediated immunosuppression. Spectrum of antimicrobial activity. The addition of cerium nitrate to silver sulfadiazine probably gives Gram-positive and Gram-negative organisms and fungus superior antimicrobial activity. 

 Clinical use. The clinical use of cerium nitrate-silver sulfadiazine is identical to that of silver sulfadiazine alone. The drug combination produces an adherent eschar that provides satisfactory wound coverage until tangential excision can be carried out. Clinical trials showed no difference in mortality between silver sulfadiazine used alone and with the addition of cerium nitrate. 

 Adverse effects. The adverse effects of cerium nitrate silver sulfadiazine are similar to those seen with silver sulfadiazine alone [**2,5,11**]. 

 Mafenide (Sulfamylon 10%). With its excellent antimicrobial activity and the best eschar penetration of any agent, mafenide was dropped from general clinical use because of its severe side effects, especially when applied in large areas. Mechanism of action. Mafenide appears to act in the bacterial cellular metabolism. Spectrum of antimicrobial activity. In topical application, mafenide is bacteriostatic against Gram-positive and Gram-negative bacteria. However, it has limited action against S. aureus and fungus. The development of resistant organisms has not been reported. Clinical use. Mafenide is used as an adjunct in the treatment of bacterial invasion in second- and third-degree burns to prevent septicemia caused by susceptible organisms. It also efficiently penetrates cartilage, which makes it an excellent choice for use in burned ears and noses. Adverse effects. Pain or a burning sensation following mafenide application is the most frequently reported adverse effect. Mafenide is a strong carbonic anhydrase inhibitor and its use leads to alkaline diuresis, which can cause acid base abnormalities. It also inhibits epithelial regeneration [**1,8,11**]. 

 Silver nitrate solution 0.5 % (Moyer et al., 1965) Mechanism of action. The effects of silver nitrate may result from silver ions readily combining with sulfhydryl, carboxyl, phosphate, amino, and other biologically important chemical groups. Spectrum of antimicrobial activity. Silver nitrate is a broad-spectrum agent, bacteriostatic, at a concentration of 0.5%; development of resistance to the silver ion is distinctly uncommon. 

 Clinical use. Silver nitrate 0.5% is effective in prophylactic use in second- and third-degree burns; however, it does not penetrate burn eschar, and needs bulky and frequent dressing changes, which limits its use. 

 Adverse effects. Silver nitrate is prepared with distilled water resulting in an extremely hypotonic solution leading to electrolyte imbalance. Methemoglobinemia is another potential complication, owing to the reduction of nitrate to nitrite by bacteria [**1**]. 

 Nitrofurazone 

 Mechanism of action. It appears that the drug acts by inhibiting bacterial enzymes involved in carbohydrate metabolism. Organic matter inhibits the antibacterial action of nitrofurazone. 

 Spectrum of antimicrobial activity. Nitrofurazone has a wide spectrum of activity against Gram-positive and Gram-negative bacteria. However, HoIt is not particularly active against most strains of P. aeruginosa and does not inhibit fungi or viruses. S. aureus and E. coli can develop resistance to nitrofurazone. 

 Clinical use. Nitrofurazone is topically used as adjunctive therapy in patients with second- and third-degree burns. With good eschar penetration, it can be used in the treatment of invasive burn wound infections with sensitive agents. The drug presents some advantages for the ambulatory patient. Tissue granulation begins sooner and separately crusts more rapidly. 

 Adverse effects. Allergic contact dermatitis is the most frequently reported adverse effect, occurring in approximately 1% of the patients treated. The polyethylene glycols present in nitrofurazone ointment (but not in cream) can be absorbed through denuded skin and may cause renal impairment [**2,11**]. 

 Chlorhexidine 

 Mechanism of action. Chlorhexidine acts by destroying the bacterial cellular wall and precipitation of cellular content. 

 Spectrum of antimicrobial activity. Chlorhexidine has a broad spectrum of antimicrobial action, but some Pseudomonas and Proteus species develop resistance. 

 Clinical use. Several preparations of chlorhexidine have been clinically used in moderate burn injuries and are the subject of continuing investigations. The addition of 1% chlorhexidine digluconate to 1% silver sulfadiazine increases the agents’ antibacterial effectiveness. 

 Adverse effects. The most frequent adverse effects of chlorhexidine digluconate are local pain and ototoxicity [**10**]. 

 Povidone-iodine 

 Mechanism of action. Povidone iodine acts by destroying microbial protein and DNA. 

 Spectrum of antimicrobial activity. This drug has excellent in vitro antimicrobial activity but is inactivated by wound exudate. Clinical use. Povidone iodine has not been proved useful as a topical antimicrobial treatment for burn patients. 

 Adverse effects. Systemic absorption of iodine, with resulting renal and thyroid dysfunction [**11**]. 

 Table 1 presents a summary of the antibacterial activity of the above-mentioned drugs.

**Table 1:**
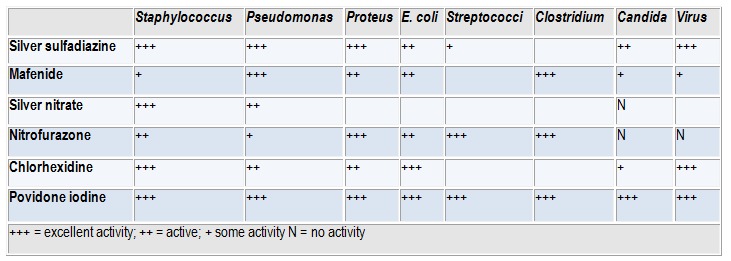
Antimicrobial activity [**10**]

 Norfloxacin: because of its broad spectrum of antimicrobial action, norfloxacin and its silver salt were formulated in a topical cream base. However, they warrant further development as topical anti-infective agents for use in treating burn patients [**2,6**].

 Mupirocin: Methicillin-resistant S. aureus (MRSA) strains have become increasingly prevalent as nosocomial pathogens, especially in burn wounds. Both in vitro and in vivo mupirocin has proved to be highly effective in the treatment of MRSA infections. The safety and efficacy of mupirocin ointment in burns exceeding 20% TBSA need to be established. The use of intranasal mupirocin ointment appears to reduce the risk of infection among patients during MSRA-related outbreaks [**3,13**].

 Sodium hypochlorite: the use of sodium hypochlorite as a topical agent was abandoned as a topical agent because of its basic pH, which causes pain and has a low antimicrobial effect. This problem may be solved by using hypochlorite [**3**].

 Other agents: Many other antimicrobial agents are used in the local therapy of the burn patient. The clinical usefulness of some of these requires further study. The following are some of the most cited in literature:

 Gentamicin

 Gentamicin cream should be reserved for treating patients with wounds infected by gentamicin-sensitive P. aeruginosa and patients allergic to sulfa drugs, because of the appearance of gentamicin-resistant strains.

 Bacitracin

 Most clinical use of bacitracin is in the prophylaxis of Gram-positive bacteria infection in open areas. The addition of neomycin and polymyxin B expands the antimicrobial action to Gram-negative bacteria. A physiological pH. A freshly prepared 0.1% NaOCl solution decontaminates skin colonized with S. aureus, C. albicans, and P. aeruginosa within 10, 20, and 30 min, respectively. There is no report of microbial resistance to hypochlorite. More studies need to be done before the clinical use is possible. Hypochloride has the additional advantages of reducing edema and of having no effect on granulation and epithelialization.

 The incidence of invasive infection and overall mortality was significantly reduced after the introduction into clinical practice of topical burn wound antimicrobial agents. The drug of choice for prophylaxis in most burn patients is silver sulfadiazine. The addition of cerium nitrate appears to enhance bacterial control in large burns and the addition of other drugs such as chlorhexidine and norfloxacin seems to reduce the emergence of bacterial resistance. In selected clinical situations, mafenide, nitrofurazone and mupirocin may be useful. New agents and new combinations of existing agents are to be seen in the literature, but their clinical efficacy has to be confirmed [**3,6**].

 Dressing with a slow release of active principle:

 Acticoat: consists of two layers of a silver-coated, high-density polyethylene mesh, enclosing a single layer of an apertured non-woven fabric of rayon and polyester. The three components are ultrasonically welded together to maintain the integrity of the dressing in use. Silver is applied to the polyethylene mesh by a vapour deposition process, which results in the formation of microscopic "nanocrystals" of metallic silver. In this nanocrystalline form, metallic silver exhibits pronounced antibacterial activity against a wide range of Gram-positive and Gram-negative bacteria including strains resistant to many types of antibiotics. It is also effective against clinically important strains of yeasts and fungi. Indications: Acticoat is used as an antimicrobial barrier layer for partial and full-thickness wounds such as burns, donor sites and graft recipient sites that are judged to be at risk from infection. Contra-indications: Acticoat is contra-indicated in patients with known hypersensitivity to any of the components of the product. If signs of a sensitivity reaction develop during use, treatment should be discontinued. No safety issues associated with the use of Acticoat have been identified to date. I recommend that the dressing is left in place for a maximum of three days, although on very heavily exuding wounds, it may be necessary to be replaced more frequently [**11**].

Prontosan: ready-to-use solution containing polyhexanide and undecylenamidopropyl betaine is used for cleansing, moistening and keeping wounds and wound dressing moist, releases fibrin coatings and residues from wound coatings in a way that protects the tissue.

**Fig. 1:**
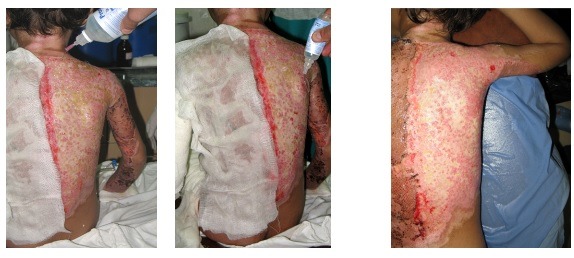
Local direct application from the practical bottle of Prontosan

**Fig. 2:**
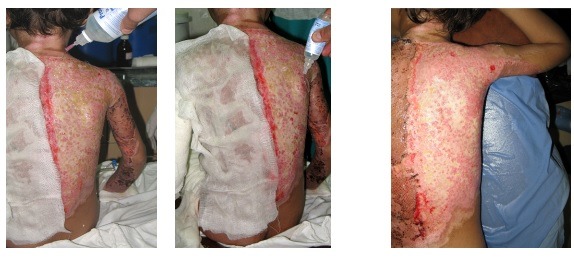
Local direct application from the practical bottle of Prontosan

**Fig. 3:**
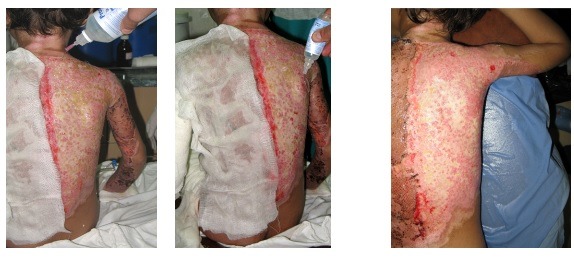
Aspect after 7 days of local treatment with Prontosan

 A good alternative for escharectomy-grafting seems to be enzymatic debridement.

 Selective and rapid enzymatic debridement preserves the non-injured tissue, in particular the dermis with its collagen and epithelial elements. When provided with proper conditions for epithelial proliferation and propagation, the newly debrided collagen bed allows spontaneous and rapid re-epithelization, generally in 2-3 weeks. Surgical debridement procedure could be replaced in the future by an efficient enzymatic debridement that results in less patient trauma and more rapid healing [**9,11**]. 

**Fig. 4:**
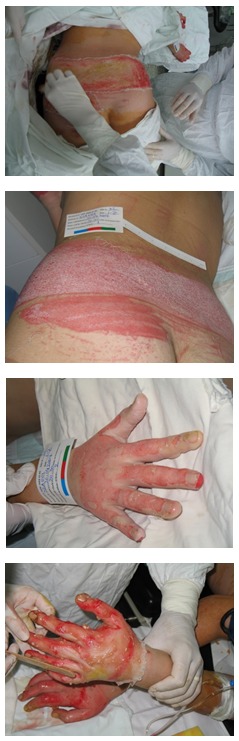
Enzymatic debridement – Pre and Post treatment

 In case of extensive burns, thus a problem of great importance, what is important is finding appropriate methods of skin covering, the ultimate goal being definitively covering with the least sacrifice for the unaffected tissues, obtaining good functional and aesthetical results. A large series of options are available; those which are frequently used in our clinic will be mentioned and detailed [**11,13**].

 Definitive skin covering

 Autografting: the graft represents the ideal solution of definitive skin covering (easy harvesting, exceptional covering quality, reduced costs) [**14,16**]. 

 Autografts: can be full thickness (containing epidermis and derma in totality, including annexes) or intermediary (epidermis and a variable thickness from derma).

**Fig. 5:**
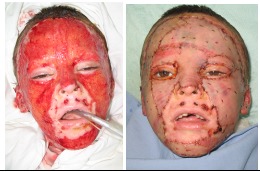
Autografts for covering facial anatomic units

 Full thickness grafts, the first type described, assure an excellent cosmetic outfit but have a more difficult integration. The available skin surface is limited by the necessity to close the donor zone through suture. In the situation in which the covering of larger surfaces with full thickness skin grafts is necessary, as in the case of covering the burns of the face, the donor zone can be increased with preoperative expansion techniques [**20**].

 Intermediary skin graft is now the most frequently used form of tissue transplant in modern plastic surgery. It has the advantage of being harvested from wide areas and of having a good integration, but it is predisposed to contraction and hypertrophic scars, especially in children. Their donor zone heals through spontaneous insular epithelization starting from the epithelial rests from the annexes. The expansion 1:1,5 up to 1:9 is used especially in extensive burns, assuring an improved drainage of the graft beds, but the cosmetic appearance after epithelization is deficitary. Non-expanded grafts can be used in extensive burns up to 55% and are used in burns larger than 55% only for the face and hands (due to the superior cosmetic result) [**18**].

**Fig. 6:**
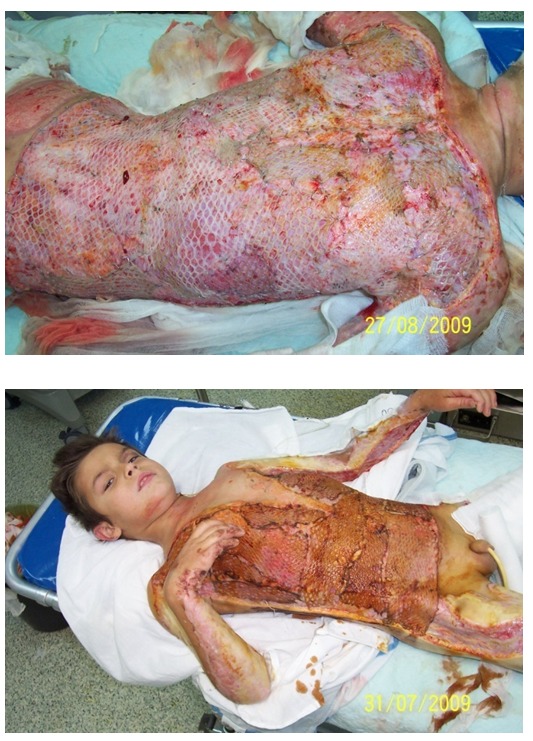
Extensive Autografting

 The limits of the method regards extensive burns, in which the potential donor skin reserves are insufficient, new methods of temporary or definitive (dermo-epidermal cultures of keratinocytes in the presence of fibroblasts) skin covering being needed [21,22]. 

 Alternative methods for definitive skin covering are dermal matrix, composites materials, autologous epidermal culture.

 The concept of dermal matrix-cellular epidermal component as a alternative method of definitive skin covering is of wide interest. The autologous epidermal culture solves only the problem of epidermal component of skin loss as the tridimensional dermal matrix-autologous epidermal culture solves dermal and epidermal loss too. Ideally, this kind of alternative covering material must be nonimmunologic, nontoxic, antibacterial, to prevent wound drying, firm adhesion to the receptor site, to permit ulterior growing of the pediatric patients, to stop the forming of granulation tissue. Dermal matrix: there are various types of dermal matrix. The differences between them are mainly: sponginess, thermal stability, degree of contracture, degree of denatured proteins [**15,19**]. 

 Integra acellular synthetic material is composed of two layers.

**Fig. 7:**
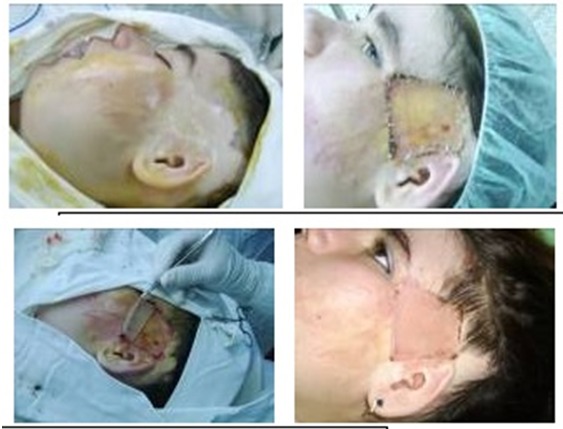
INTEGRA and 21 days later autograft for covering the facial defect

 The superior sylastic layer has a type of sponginess, which prevents bacterial colonization and prevents wound drying. On top of that is a silicone membrane almost similar to the normal epidermis that must be replaced after three weeks with autografts or autologous epidermal culture.

 The inferior layer is composed by bovine collagen coprecipitate and condroitin-6-sulfate and allows the migration of endothelial cells and fibroblasts. The result is a new dermal structure.

 Composites materials: allograft transplant combined with autologous epidermal culture, collagen matrix combined with autologous epidermal and fibroblastic cultures. The microscopic analyses showed dermal-epidermal renewal after two weeks and after three months final skin aspect, a low inflammatory reaction and new collagen reserves.

 Autologous epidermal culture

**Fig. 8:**
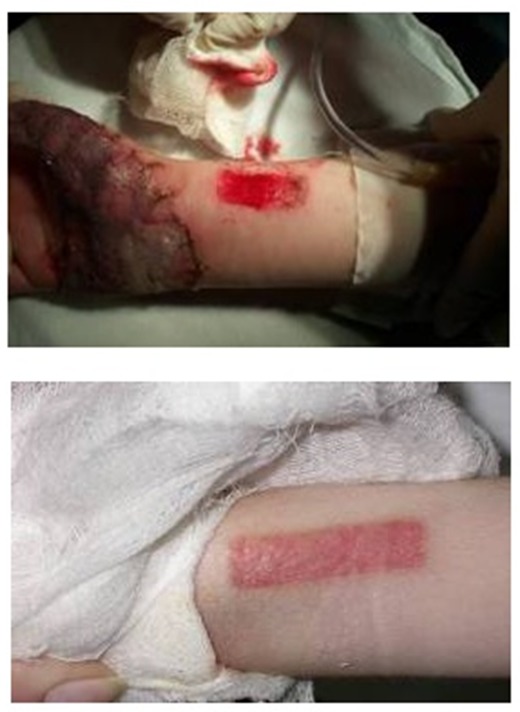
Day 1 and 4 after epidermal culture

 Temporary skin covering 

 In the extensive deep burns, in which the skin reserves of the patient are insufficient to achieve an efficient skin covering, we temporarily use skin substitutes, to allow the organism to regenerate its own reserves. Skin substitutes may be natural such as allografts, xenografts, amniotic membranes or synthetic and semi-synthetic such as bilayered synthetic structures, composite materials based on collagen (BIOBRANE)[**11,17,18**].

 The qualities of an ideal skin substitute: firm adhesion to the receptor site, prevents wound drying, antimicrobial barrier, reduces pain, durability, flexibility, non-toxicity.

 Allograft represents a tissue transfer from one individual to another of the same species.

 Allografts were first transplanted organs and gained knowledge in this field, constituting the fundamentals of modern transplant immunology. Because of the marked skin antigenicity, only autografts are not rejected (and in rare cases isografts). Immunosuppression from the major burns can delay allografts reject up to several weeks. At this rate, allografts are utilized with good result, with or without autografts, in the extensive burns. 

 "Sandwich" technique overlaps a layer of medium expanded allograft over intermediary largely expanded autografts, which proved to have superior results to autografts use only.

 Allograft advantages: bilayered structure identical to the destroyed tissue, possible revascularization, dermal structures present, drying prevention, promotes dermal receptive area development, reduces liquid and cellular losses, favors early rehabilitation, noble elements protection. Allografts disadvantages: rejection after 2-4 weeks, risk of contamination (considerably decreased if preserved in glycerol 85%) [17,21].

**Fig. 9:**
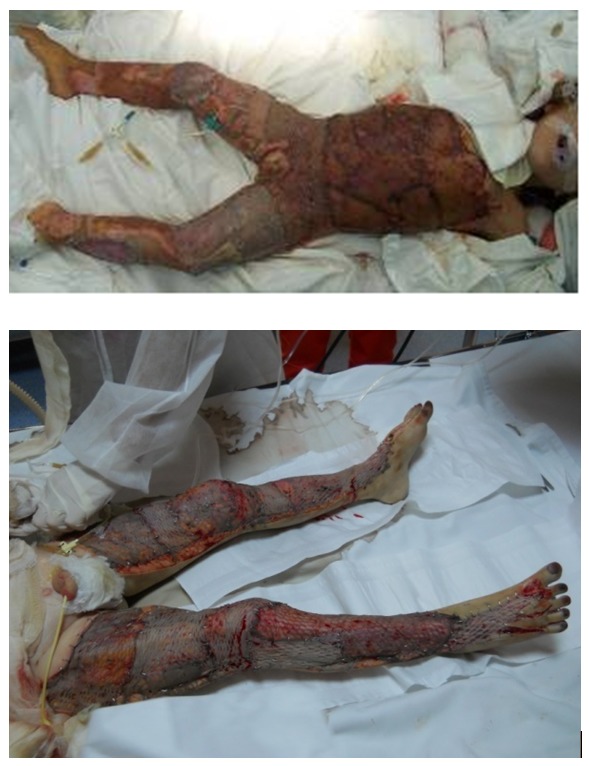
Combined covering methods - lower limbs covered with autografts and xenografts sandwich; trunk covered with autografts

 Xenografts represent transfers of tissue from one species to another. Pig xenografts have been used either as temporary dressing or as "sandwich"-like structures, covering largely expanded autografts in the extensive burns. The unique adherence qualities of pig xenografts, scientifically explained through the biologic process of binding fibrin-elastin, are responsible for the antibacterial effect, too. The use of xenografts is also beneficial because it stimulates formation of granulation tissues and prepares the wound for future autografting. The utilization of xenografts was extended, not merely for covering large burns before grafting, but also for temporary covering of exposed vessels and tendons, leg ulcers, donor zones.

 Xenografts have some advantages: can be obtained easier than allografts, are bioactive (collagen matrix permits the adherence to the wound) and also some disadvantages: they cannot be integrated, they suffer a reject process, contamination risk [**21,22**].

 In 2006, xenografts were used for a large number of cases of extensive burns in our clinic. Following this experience, the next conclusions were reached: freshly harvested xenografts are more efficient than those cryopreserved; xenografts adhere to the receptor bed which was freshly excised and with a reduced degree of contamination, constituting a biologic dressing of good quality for 7-10 days, the process of rejection occurring after 10-14 days. However, the surgical excision followed by autografting is preferred, xenografts near autografts do not influence negatively the evolution of neighboring autografts, the subjacent formed dermal matrix has a good quality, favorable for autografting, the expansion of the xenografts is preferred, thus preventing subjacent exudates accumulation and infection.

 Amniotic membranes are used as the allografts. They contain fibronectin, collagen and are foreseen with a fine epithelial layer which functions like the barrier of the epidermis. Adherence to the wound is reduced. They have some advantages such as biologic barrier, easy to apply and remove, though transparency permits the visualization of subjacent tissues and also some disadvantages being difficult to obtain, remake and store, has to be replaced frequently (2-3 days), they quickly disintegrate, have high risk for infections (bacterial, viral) [**14,15**].

 Biobrane is a biosynthetic skin substitute first developed in 1979 and was modified since then, being constructed of an outer silicone film (the epidermal analog), with a nylon fabric partially imbedded into the film. Collagen (porcine type 1) is incorporated both in silicone and nylon components by being chemically cross-linked. The collagen peptides on the nylon bind to the wound surface fibrin and collagen resulting in the initial adherence (dermal analog). Also, small pores are present in the structure to allow the drainage of exudate and also provide permeability to topical antibiotics. Clinical indications are: superficial to mid-partial thickness burns (once debrided of nonviable tissue), excised burn wound with or without meshed autografts (need viable bed), donor sites, partial thickness skin slough disorders [**12,13**].

## Results

From 1298 extensive burned patients admitted in our clinic during 2006-2013, there were 804 extensive scalds, 260 extensive flame burns, 104 electric injury extensive burns, 65 extensive contact burns, and 65 victims of collective accidents. Sex ratio was 1,6/1 male patients over female patients. Referring to age distribution, regarding extensive scalds the age group 1-4 years old is more frequent; for flame burns 3-15 years old, electric injuries 7-17 years old; contact burns 1-3 years old, collective accidents all ages.

 Treatment of superficial and partial superficial burns included symptomatic treatment, topical antibacterial dressings (silver sulfadiazine, mafenide acetate), correct positioning, and early mobilization. The objective is spontaneous epithelization in 8-10 days. 

 Treatment of partial deep and full thickness burns included decompression incisions if vascular insufficiency, early excision and grafting. In extensive burns, facial and hand burns have surgical priority compared to other regions. Not all the patients from the study required surgery. 84% of the admitted extensive burns healed after proper general and local treatment.

**Fig. 10:**
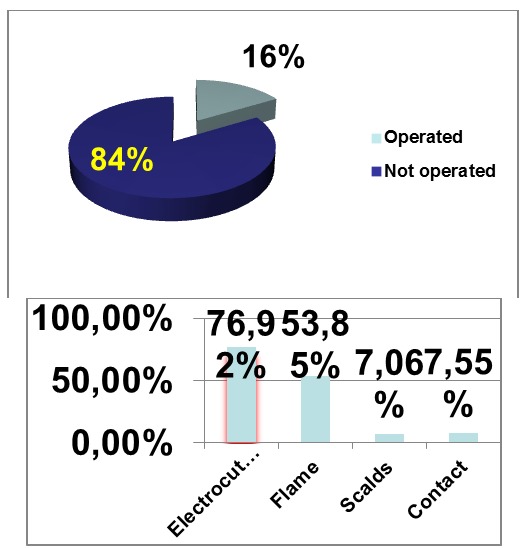
Ethiology related with surgery procentage

**Fig. 11:**
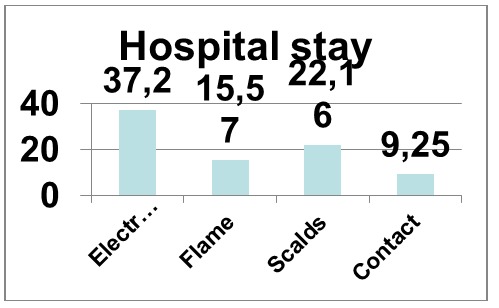
Etiology related hospital stay

 Average hospital stay was highest for electrocutions (37,2 days) followed by scalds (22,16 days), flame burns (15,57 days) and contact burns (9,25 days).

## Conclusions 

The general philosophy of the burns has to be applied on each case and meticulous local care is an essential aspect.

 Highly standardized therapeutic protocols, specialized teams and good technical conditions, can considerably improve the results for burn patients.

 All local burn treatment methods must be modulated in connexion with the particularities of every burn lesion and every patient.
